# Prickly Ash Seeds Improve the Ruminal Epithelial Development and Growth Performance of Hu Sheep by Modulating the Rumen Microbiota and Metabolome

**DOI:** 10.3390/microorganisms12112242

**Published:** 2024-11-06

**Authors:** Qiao Li, Yi Wu, Xingcai Qi, Zilong Liu, Chunhui Wang, Xueyi Ma, Youji Ma

**Affiliations:** 1 College of Animal Science and Technology, Gansu Agricultural University, Lanzhou 730070, China; liqiao1109@163.com (Q.L.); wy1593050417@126.com (Y.W.); 18794226760@163.com (X.Q.); lzl107332202076@163.com (Z.L.); chwang5522@163.com (C.W.); maxueyi0303@163.com (X.M.); 2 Gansu Key Laboratory of Animal Generational Physiology and Reproductive Regulation, Lanzhou 730070, China

**Keywords:** prickly ash seeds, rumen microbiome, metabolites, rumen development, growth performance, sheep

## Abstract

It is known that the addition of feed rich in bioactive components to animal diets will affect rumen fermentation parameters and flora structure. However, research on the regulatory effects of prickly ash seeds (PASs) during rumen development or on the rumen microbiome and its metabolites in sheep is limited. The current study was designed to explore the effects of PASs on sheep rumen development and growth performance using metagenomics and metabolomics. Eighteen 3-month-old Hu lambs were randomly allotted to three different dietary treatment groups: 0% (basal diet, CK), 3% (CK with 3% PAS, low-dose PAS, LPS), and 6% (CK with 6% PAS, high-dose PAS, HPS) PASs. The lambs were slaughtered to evaluate production performance. Our results showed that dietary PAS addition improved the average daily gain and reduced the F/G ratio of the experimental animals. Additionally, the height and width of the rumen papilla in the treatment groups were significantly higher than those in the CK group. The fermentation parameters showed that the levels of acetate and butyrate were significantly higher in the LPS group than in the CK and HPS groups. The propionate levels in the HPS group were significantly higher than those in the CK and LPS groups. Metagenomics analysis revealed that PAS dietary supplementation improved the abundance of Clostridiales and Bacteroidales and reduced the abundance of *Prevotella*, *Butyrivibrio*, and *Methanococcus*. Metabolomic analyses revealed that increased metabolite levels, such as those of serotonin, L-isoleucine, and L-valine, were closely related to growth-related metabolic pathways. The correlations analyzed showed that papilla height and muscular thickness were positively and negatively correlated with serotonin and L-valine, respectively. Average daily gain (ADG) was positively and negatively correlated with L-valine and several *Prevotella*, respectively. In addition, muscular thickness was positively correlated with *Sodaliphilus pleomorphus*, four *Prevotella* strains, *Sarcina_sp_DSM_11001*, and *Methanobrevibacter_thaueri.* Overall, PAS addition improved sheep growth performance by regulating beneficial microorganism and metabolite abundances, facilitating bacterial and viral invasion resistance.

## 1. Introduction

The large-scale and extensive development of the livestock industry in China, to improve the economic benefits of farmers, has led to the shortage of feed resources, which has become one of the main factors restricting livestock industry development. Therefore, to ensure the long-term sustainability of animal husbandry and to reduce competition for human food resources, it is important to develop alternative and unconventional feed resources. China has a large number and variety and wide distribution of unconventional feed resources, and the development and utilization of these can provide new feed sources for the livestock industry. Some unconventional feed resources are derived from the by-products of functional plants that have antibacterial, antioxidant, and growth-promoting effects; moreover, they contain active ingredients, such as flavonoids and polyphenols, which can also function as antibiotics to a certain extent, and are therefore considered safe, non-polluting, and green products [1,2]. The application of green plants and their extracts in ruminants has a positive influence on rumen fermentation, immune functions, and animal products. For example, adding *Cistanche deserticola*, *Astragalus membranaceus* root, and *Alhagi camelorum* Fisch polysaccharides to sheep feed can improve rumen fermentation, growth performance, immunity, and antioxidant capacity and modulate the rumen flora structure [3,4,5]. Therefore, it is important to develop functional unconventional feed and apply it to animal production to alleviate the shortage of feed resources and improve the production performance and overall health of sheep.

Prickly ash (PA) (*Zanthoxylum bungeanum* Maxim) is a deciduous tree belonging to the Rutaceae family. It has a long planting history in China and is widely distributed in the Gansu, Sichuan, Hebei, Shanxi, and Shandong provinces [6,7]. Notably, prickly ash seeds (PASs) account for 60–70% of the total weight of PA fruit, making it the main processing by-product in the PA industry. PASs are rich in nutrients and can be used as a good alternative feed resource [8]. In addition, PASs and their processed products contain polyphenols, flavonoids, alkaloids, and other biologically active ingredients, which are widely used in the food processing and livestock feed industries. Mu et al. found that the total polyphenol content of PASs ranges from 72.83 to 138.84 mg/g and that the flavonoid content ranges from 29.78 to 57.56 mg/g after measuring the total active polyphenol and flavonoid constituents in solutions of different polarities [9]; moreover, both compounds were found to have functional properties, such as antioxidant, anti-inflammatory, and intestinal immune activities [10,11]. Recent studies have shown that the addition of flavonoid and their extracts from different sources on dairy cows and sheep can improve rumen fermentation parameters and reduce methane and ammonia by changing the structure of rumen microbial communities [12,13]. At the same time, the addition of green tea polyphenols and polyphenol extracts to animal diets can enhance the integrity of the intestinal barrier and improve rumen fermentation parameters by changing the structure of gastrointestinal tract microbial flora [14,15]. These results indicate that dietary supplementation with plants or extracts rich in polyphenols and flavonoids can alter the intestinal microflora and improve intestinal barrier functions.

Currently, the application of PASs in animal husbandry only affects animal growth and production performance. For example, in pigs, replacing a certain proportion of corn with PASs in feed can alter the fatty acid (FA) composition of muscle, affecting meat quality [16]. Tian et al. found that adding oil-rich PASs to the diet of Jian carp did not adversely affect their growth or health status [17]. Our previous study showed that the addition of PASs to sheep feed affects the apparent digestibility of nutrients, intestinal development, immune functions, and digestive enzyme activity [18]. Although PASs have been applied in animal production, owing to differences in the digestion and absorption mechanisms of nutrients in the gastrointestinal tracts among fish, monogastric animals, and ruminants, the mechanisms underlying their effects on animals are also different. However, few studies have focused on the regulatory effects of PASs during rumen development or on microbial structural characteristics, metabolites, or metabolic pathways in sheep.

Given that PASs are rich in bioactive components, we hypothesized that their addition to the diet would have a positive effect on growth performance in sheep mediated by regulating changes to the rumen microflora and metabolites. Therefore, the purpose of this study was to investigate whether the dietary supplementation of PASs rich in flavonoids and polyphenols had a positive effect on the rumen development and growth performance of sheep mediated by regulating the interaction between microorganisms and microbial metabolites in the rumen. The results of this study are expected to provide a theoretical basis for the application of PASs and their by-products in ruminants.

## 2. Materials and Methods

### 2.1. Ethics Statement

The present study was approved by the Ethics Committee of the Gansu Agricultural University (GSAU-Eth-ASF2022-008).

### 2.2. Preparations of Diets

PASs were collected between August and September 2021 at a planting base of prickly ash, between 103°10′ and 44′ east longitude and 35°30′ and 36′ north latitude, Dongxiang Autonomous County, China. The nutritional content of PASs is presented in Supplementary Table S1. We analyzed the metabolites in PASs using high-performance liquid chromatography–mass spectrometry; among the metabolites, flavonoids and phenols accounted for 10.97% and 4.52%, respectively (Supplementary Figure S1). The basal diet was prepared according to the “Feeding standard of meat-producing sheep and goats (NYT816-2021)” in China (Supplementary Table S2).

### 2.3. Animals, Feeding Management, and Experimental Design

Eighteen 3-month-old Hu lambs (25.66 ± 3.03 kg body weight) were randomly allotted to three different dietary treatment groups. In the three dietary treatments, 0% (basal diet, CK), 3% (CK with 3% PAS, low-dose PAS, LPS), and 6% (CK with 6% PAS, high-dose PAS, HPS) PASs were used. The addition of PASs in the LPS and HPS groups replaced an equivalent amount of roughage, ensuring that all experimental diets had the same nitrogen and energy levels. The experiment period lasted 100 days, including a 10-day adaptation period and a 90-day experimental period. During the feeding period, a total mixed ration was provided to Hu lambs at 08:00 and 18:00 every day with the feed supply adjusted according to 5~10% of the residual feed. All lambs had ad libitum access to food and water. During this experiment, the amount of feed offered daily and the quantity remaining in the feeder the next morning were accurately recorded. The lamb weights were measured on the first and last days of the experimental feeding period before the morning meal. The daily weight changes and feed intake were used to calculate the average daily weight gain (ADG), average daily intake (ADFI), and feed-to-gain ratio (ratio of ADFI to ADG, F:G). After slaughter, hot carcass weight was recorded immediately.

### 2.4. Sample Collection

On the last day of the experiment (d 90), after fasting for approximately 12 h, all lambs were slaughtered by exsanguination after euthanasia by electrical stunning. Subsequently, the rumen epithelial tissue was cut from the same dorsal sac of the sheep rumen and fixed in 4% paraformaldehyde for histomorphological analysis. The rumen epithelial morphology parameters were observed after scanning using Case Viewer 2.3 software (3DHistech, Budapest, Hungary), and the thickness of the rumen muscle layer, papillary height, and papillary width were calculated. Then, rumen fluid was collected and strained through 4 layers of gauze; some of the fluid was stored at −20 °C for the determination of volatile fatty acids (VFAs), pH, and ammonia-N, and another portion was frozen in liquid nitrogen and stored at −80 °C for rumen microorganism and metabolite determinations.

### 2.5. Determining Fermentation Parameters

The rumen fluid pH was measured using a portable pH meter (Sartorius PB-10, Sartorius, Göttingen, Germany) during fluid collection. Frozen rumen fluid samples were thawed at 4 °C and centrifuged at 5400 rpm for 10 min, and the supernatant was collected for the subsequent analysis. A colorimetric method was used to detect NH_3_-N, as described by Wang et al. [19]. The VFA concentrations were tested as previously described [20]. Briefly, we mixed 1 mL supernatant and a 0.2 mL 25% metaphosphate solution, containing 2 EB as an internal standard, and uniformly mixed them in a new centrifuge tube. The mixture was placed in an ice bath for 30 min and centrifuged for 10 min with 10,000× *g* in 4 °C conditions. After filtering through a 0.22 μm organic pinhole filter membrane, the filtrate was stored in a sample bottle at 4 °C for testing. VFA concentrations were measured using gas chromatography (Agilent, Palo Alto, CA, USA). More details about the VFA determination can be found in Shi et al. [21].

### 2.6. Metagenome Sequencing and Data Processing

Total genomic DNA was extracted from rumen contents according to the protocol of the DNA extraction kit (Magen, Guangzhou, China), and the concentration and quality of the extracted DNA were determined using the NanoDrop^®^ ND-2000 spectrophotometer (NanoDrop Technologies, Thermo Scientific, Waltham, MA, USA). Genomic DNA was randomly interrupted into small fragments of about 300 bp using Covaris M220 (Gene Company Limited, Hong Kong, China), and metagenomic libraries were constructed using TrueSeq DNA PCR-Free Library Prep Kits (Illumina, San Diego, CA, USA). After the libraries were tested for qualification, they were sequenced using the Illumina HiSeq X-ten platform (Illumina, San Diego, CA, USA) with a PE150 strategy.

Sickle (version 1.33, https://github.com/najoshi/sickle, accessed on 10 June 2024) was used to process and control sequencing data quality. Briefly, the 3′- and 5′-end of reads, low-quality reads (quality scores < 20), short reads (<100 bp), and “N” records were removed. Owtie 2 (V 2.3.4.1) [22] was used to align the reads to the sheep reference genomes (GCA_000298735.1), and hits associated with the reads and their matched reads were removed. The filtered reads were assembled de novo using https://github.com/voutcn/megahit, accessed on 12 June 2024. Overlapping sequences with a length of >300 bp were selected as the final assembly results and used for further gene prediction and annotation. MetaGene (http://metagene.cb.k.u-tokyo.ac.jp/, accessed on 12 June 2024) was used to predict the open reading frames [23]. The non-redundancy assembled contigs were clustered using CD-Hit with 95% identity (http://www.bioinformatics.org/cd-hit/, accessed on 17 June 2024) [24]. The original sequencing reads from each sample were mapped to representative sequences to estimate gene abundances using SOAPaligner (http://soap.genomics.org.cn/, accessed on 18 June 2024) [25].

### 2.7. Taxonomic and Functional Analysis

DIAMOND v.0.9.30 (https://github.com/bbuchfink/diamond, accessed on 5 July 2024) was used to perform a taxonomic assessment of the rumen microbiome based on the NCBI Non-Redundant database [26]. The metagenome functions were annotated using the blast function Diamond, against the Kyoto Encyclopedia of Genes and Genomes (KEGG) database (*E*-value ≤ 1 × 10^−5^) [27]. Carbohydrate active enzyme (CAZyme) annotation was performed using USEARCH (http://www.drive5.com/usearch/, accessed on 10 July 2024) [28].

### 2.8. Metabolomic Analysis

The experimental metabolomic analysis process included sample extraction and analysis, metabolite identification and annotation, data quality control, and statistical analysis, all of which were performed at Wuhan MetWare Biotechnology Co., Ltd. (Wuhan, China), following their standard procedures [29]. The samples were analyzed using an LC-ESI-MS/MS system (UPLC, ExionLC AD, https://sciex.com.cn/, accessed on 20 July 2024; MS, QTRAP^®^ 6500+ System, https://sciex.com/, accessed on 20 July 2024). The UPLC conditions comprised the parameters described previously [30]. Finally, data were acquired using multiple reaction monitoring with a triple quadrupole tandem mass spectrometer. The datasets were processed using Analyst software version 1.6.3 (Applied Biosystems, Whitby, ON, Cananda) and MultiQuant software version 3.0.3 (Absciex, Whitby, ON, Cananda). Subsequently, principal component analysis (PCA) and orthogonal partial least squares discriminant analysis (OPLS-DA) were performed to analyze the data. Based on the results of OPLS-DA, the variable importance in projection (VIP) of the obtained multivariate analysis OPLS-DA model was used to preliminarily screen metabolites in different groups. In this study, VIP > 1, FC  ≥ 1.2, or FC ≤ 0.83 was set as a criterion for differential metabolite (DM) screening.

### 2.9. Statistical Analysis

All data were analyzed using SPSS software (version 22.0; SPSS Inc., Chicago, IL, USA). Date on production performance, rumen epithelial parameters, and fermentation parameters were analyzed using a one-way ANOVA. The statistical differences were examined via Tukey’s multiple comparison tests, and statistical significance was considered at *p* ≤ 0.05. The results are presented as the mean ± standard error of the mean.

Principal coordinate analysis and Student’s *t*-tests were used to test for differences in each microbial domain between groups. Rumen microbial phyla, genera, and species were compared via Metastats analysis, with an FDR-adjusted *p* value < 0.05 considered significantly different. Linear discriminant analysis (LDA) effect size (LEfSe) analysis was used to identify the differential microbial communities between the CK and LPS groups; differences were considered significant for an LDA score > 3.0 and an FDR-corrected *p* value < 0.05 [31]. OmicShare Tools (https://www.omicshare.com/tools/Home/Soft/o2pls, accessed on 27 August 2024) were used to perform a two-way orthogonal partial least squares (O2PLS) analysis. The correlation between differentiated metabolites and microorganisms with phenotypes was determined using a Pearson correlation analysis, and Pearson correlation coefficients of r > 0.5 and r > −0.5 and a *p* value < 0.05 were considered positive and negative correlations, respectively.

## 3. Results

### 3.1. Effect of PASs on Production Performance, Rumen Epithelium Development, and Fermentation Parameters

Compared to that in the CK group, dietary supplementation with PASs improved the average daily gain (*p* < 0.05). Moreover, the F/G ratio in the CK group was significantly higher than that in the LPS group (*p* < 0.05) (Table 1).

**Figure 1:**
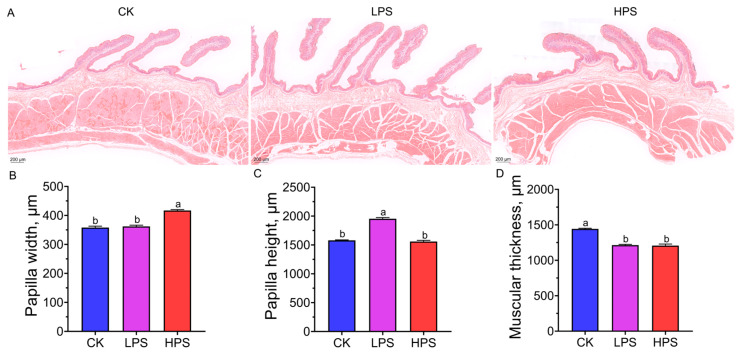
The effects of PASs on the development of the rumen epithelium. (**A**) The rumen papilla H&E staining results. (**B**) The rumen papilla width between the three groups. (**C**) The rumen papilla height between the three groups. (**D**) The muscular thickness between the three groups. a,b means significant differences with different letters (*p* < 0.05).

We performed a histological analysis of rumen epithelium development via Hematoxylin/eosin (HE) staining. The rumen papilla height in the LPS group was significantly higher than that in the HPS and CK groups (*p* < 0.05). The rumen papilla widths in the CK and LPS groups were significantly lower than those in the HPS group (*p* < 0.05), and muscle thickness in the CK group was significantly greater than that in the LPS and HPS groups (*p* < 0.05) (Figure 1). In addition, we evaluated rumen fermentation parameters in the three groups. The levels of acetate, butyrate, and valerate were significantly higher in the LPS group than in the CK and HPS groups. The propionate and acetate/propionate ratios in the HPS group were significantly higher and lower, respectively, than those in the CK and LPS groups (*p* < 0.05). However, the value of pH, NH_3_-N, iso-butyrate, iso-valerate, and total VFAs did not change significantly (Table 2).

### 3.2. Rumen Metagenome Profiling

By analyzing the data on the growth performance, rumen development, and fermentation parameters of sheep, we found that the difference between the LPS and CK groups was the most obvious. Therefore, these two groups were selected for further study. The metagenomic sequencing results showed that 80,432.24 M of data was obtained with 6702.69 M per sample. After removing low-quality reads and reads containing N bases, 80,300.31 M of data was obtained with 6691.69 M per sample, which accounted for 99.84% of the raw reads. After de novo assembly, 4,436,475 contigs were generated (369,706.25 per sample; N50 length, 1150.75 bp) (Table S3). Based on the principal coordinate analysis, a significant separation between the two groups for bacteria, eukaryotes, and viruses was noted, with no significant separation between the two groups for archaea (Figure S2). To further clarify the differences between them, we used Student’s *t*-tests to compare and analyze their microbial structure differences. The results showed significant differences in bacteria, archaea, and viruses between the two groups (adjusted *p* < 0.05); however, there was no significant difference for eukaryotes (Figure S3). Therefore, our subsequent analyses focused on rumen microorganisms including bacteria, archaea, and viruses.

### 3.3. Differences in Rumen Microbial Taxonomy

**Figure 2:**
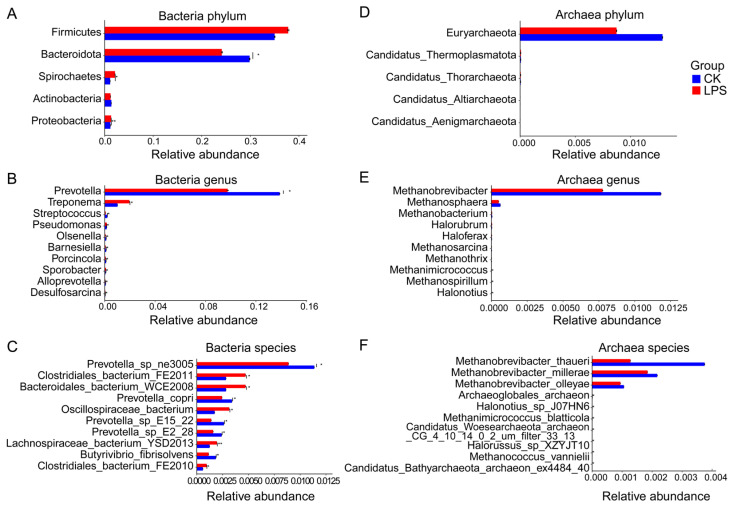
Comparison of bacterial and archaea phylum, genus, and species. (**A**) Bacterial phylum was tested by Metastats analysis. (**B**) Bacterial genus was tested by Metastats analysis. (**C**) Bacterial species were tested by Metastats analysis. (**D**) Archaea phylum was tested by Metastats analysis. (**E**) Bacterial genus was tested by Metastats analysis. (**F**) Bacterial species were tested by Metastats analysis The level of significance difference is indicated by asterisks (* (0.01 < *p* < 0.05), ** (0.001 < *p* < 0.01)).

In total, 149 phyla, 2957 genera, and 17,082 species were identified from the metagenomic sequences of bacteria, of which 10 phyla, 41 genera, and 75 species were considered relatively abundant (relative abundance > 0.1% in at least one sample). Subsequently, a comparative analysis of differential rumen microbial phyla, genera, and species between the CK and LPS groups was performed using a Metastats analysis; for the details of all species, please see Supplementary Tables S4–S6. For the top five bacteria at the phylum level, the abundance of Bacteroidota in the LPS group was significantly lower than that in the CK group; however, the abundance of Proteobacteria and Spirochaetes in the LPS group was significantly higher than that in the CK group (adjusted *p* < 0.05) (Figure 2A). In the comparison of the differential abundances of the top 10 genera exhibiting significant changes, the abundances of four genera in the LPS group were significantly higher than those in the CK group, and the abundances of six genera were significantly lower than those in the CK group (adjusted *p* < 0.05) (Figure 2B). Among the top 10 species for which abundances were significantly changed, the abundances of *Clostridiales_bacterium_FE2011*, *Bacteroidales_bacterium_WCE2008*, *Oscillospiraceae_bacterium*, *Lachnospiraceae_bacterium_YSD2013*, and *Clostridiales_bacterium_FE2010* were higher in the LPS group than in the CK group, and the abundances of *Prevotella_sp_ne3005*, *Prevotella copri*, *Butyrivibrio fibrisolvens*, *Prevotella_sp_E2_28*, and *Prevotella_sp_E15_22* were lower than those in the CK group (adjusted *p* < 0.05) (Figure 2C).

In total, 18 phyla, 117 genera, and 487 species were identified in the metagenomic sequences of archaea. Comparing archaea, the abundance of Euryarchaeota was the highest (CK, 1.29%; LPS, 0.87%) (Figure 2D). At the genus level, *Methanobrevibacter* (CK, 1.18%; LPS, 0.18%) abundance was the highest, but there was no significant difference among the groups. The abundances of *Methanospirillum* and *Methanimicrococcus* in the LPS group were significantly higher than those in the CK group, whereas the abundance of *Halonotius* was significantly lower than that in the CK group (adjusted *p* < 0.05) (Figure 2E). For species-level comparisons, the abundance of *Methanococcus vannielii* was significantly lower in the LPS group than in the CK group, whereas *Archaeoglobales_archaeon*, *Halonotius_sp_J07HN6*, *Methanimicrococcus blatticola*, *Candidatus_Woesearchaeota_archaeon_CG_4_10_14_0_2_um_filter_33_13*, and *Halorussus_sp_XZYJT10* were significantly more abundant in the LPS group than in the CK group (Figure 2F).

Further, 6 phyla, 126 genera, and 781 species were identified from virus metagenomic sequences. In comparing abundances, 13 different viruses were identified among the phyla, genera, and species. The abundance of *Phixviricota* in the LPS group was significantly higher than that in the CK group, but the abundances of most other viruses were lower than those in the CK group, indicating that the addition of PASs to the diet could reduce the prevalence of most viruses in the rumen. In summary, the addition of PASs to the diet significantly altered the composition of the rumen microbiota (Figure S4).

**Figure 3:**
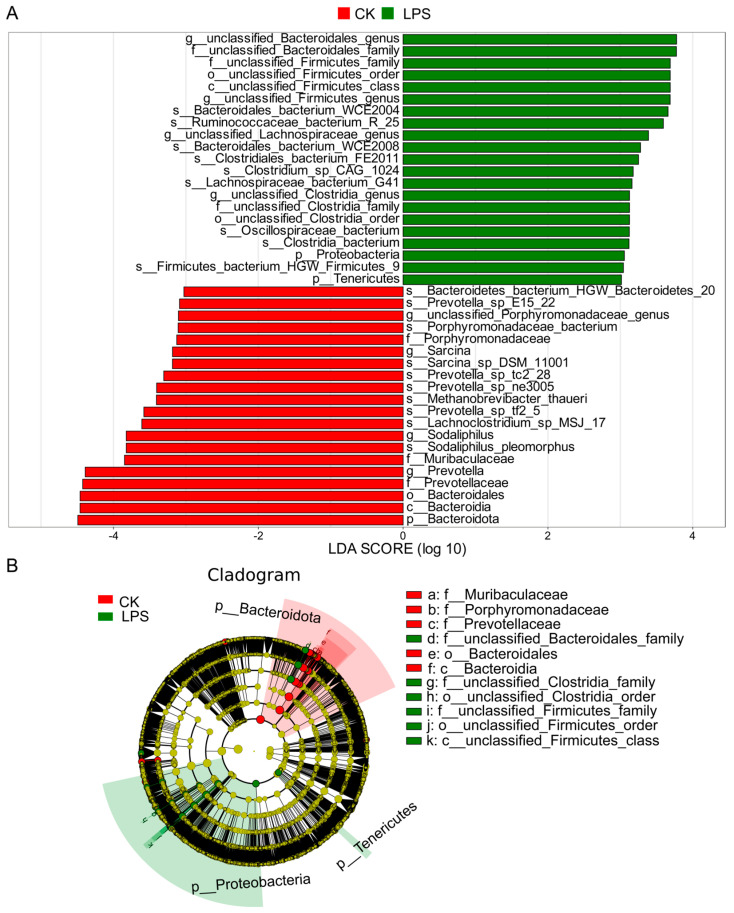
Rumen microbial biomarkers were identified by LEfSe analysis in CK and LPS groups. (**A**) LDA of LEfSe analysis (LDA > 3.0, *p* < 0.05). (**B**) Diagram of LEfSe analysis.

To further explain the differences in the microbial communities between the CK and LPS groups, we used the LEfSe Biomarker Discovery tool. For bacteria, Bacteroidota, *Prevotella*, *Prevotella_sp_E15_22*, and *Prevotella_sp_ne3005* were significantly more abundant in the CK group than in the LPS group (LDA > 3, *p* < 0.05), whereas Proteobacteria, *Clostridiales_bacterium_FE2011*, *Bacteroidales_bacterium_WCE2008*, and *Oscillospiraceae_bacterium* were significantly more abundant in the LPS group than in the CK group (LDA > 3, *p* < 0.05) (Figure 3).

### 3.4. The Functional Profiles of the Rumen Microbiome and Differential Functions

**Figure 4:**
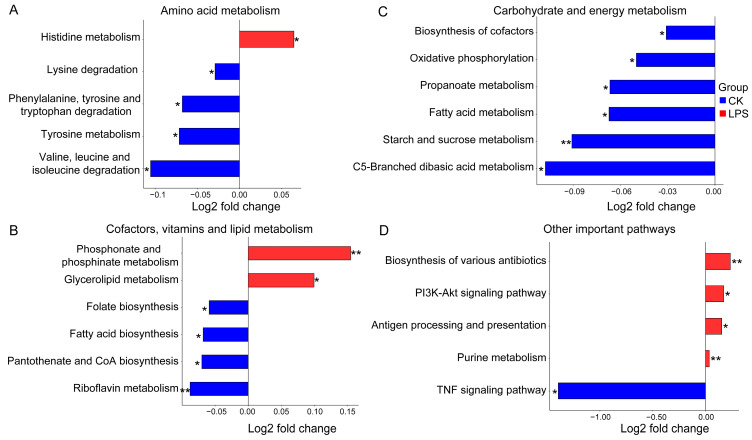
The KEGG pathway enrichment analyses of rumen flora in the CK and LPS groups. (**A**) Amino acid metabolism-related pathways. (**B**) Carbohydrate and energy metabolism-related pathways. (**C**) Cofactor, vitamin, and lipid metabolism-related pathways. (**D**) Other important metabolism pathways. The level of significance difference is indicated by asterisks (* (0.01 < *p* < 0.05), ** (0.001 < *p* < 0.01)).

**Figure 5:**
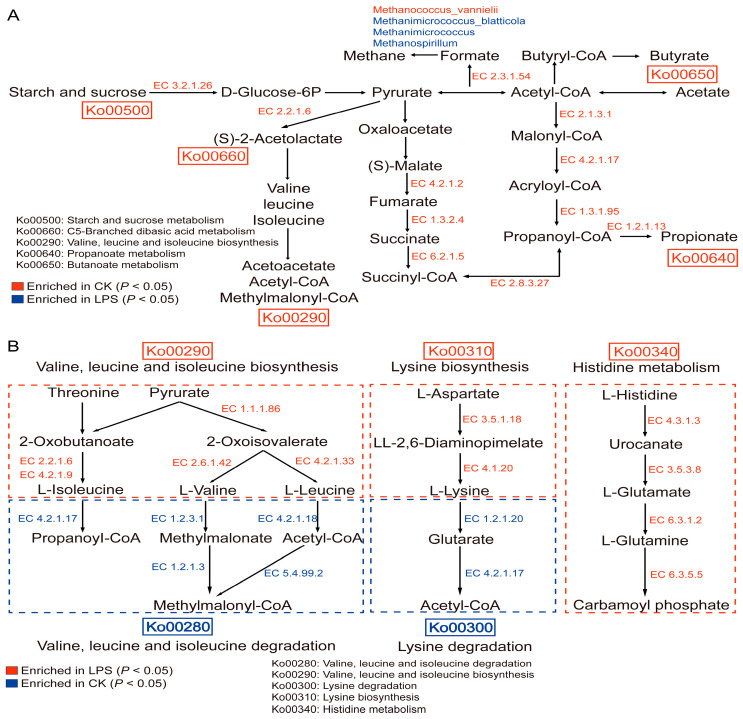
Microbial functions and species involved in (**A**) carbohydrate metabolism and (**B**) nitrogen metabolism in the rumen of CK and LPS sheep.

The functional annotation of the rumen microbiota was performed using KEGG enrichment analysis. A dimensionality reduction analysis of the Bray–Curtis distance based on species abundance revealed significant separation among the groups (Figure S5A). Metastats analysis showed that among the 20 most abundant pathways, there were 3 different pathways. Purine metabolism was significantly enriched in the LPS group, whereas the biosynthesis of cofactors, starch, and sucrose was significantly enriched in the CK group. There were no significant differences in other pathways among the groups (Figure S5B). In addition, 68 differential pathways were identified. At the first level, there were 33 metabolic pathways, 3 cellular process pathways, 6 environmental information processing pathways, 15 human disease pathways, and 11 body system pathways (Table S7). We further selected some related amino acids, carbohydrate metabolism, energy metabolism, lipid metabolism, global and overview, cofactor and vitamin metabolism, and other key pathways, which were visualized and analyzed. Among these pathways, histidine metabolism, glycerolipid metabolism, phosphonate and phosphinate metabolism, antigen processing and presentation, purine metabolism, and other pathways were significantly enriched in the LPS group, whereas oxidative phosphorylation, FA biosynthesis, and TNF signaling pathways were significantly enriched in the CK group (*p* < 0.05) (Figure 4). The products of carbohydrate metabolism, in these different pathways, can be fermented by ruminal microorganisms to produce VFAs, which can provide energy and nutrition for the growth and development of animals. In addition, since nitrogen metabolism is essential for the digestive health and nutritional utilization of ruminants, it can directly affect the digestion efficiency of feed and the production performance of animals. Therefore, we analyzed the functional roles of enzymes and genes involved in carbohydrate metabolism-related pathways (starch and sucrose metabolism, propanoate metabolism, and C5-branched dibasic acid metabolism) and nitrogen metabolism-related pathways (valine, leucine and isoleucine degradation, lysine degradation, and histidine metabolism) (Figure 5).

### 3.5. Metabolic Profile Analysis

**Figure 6:**
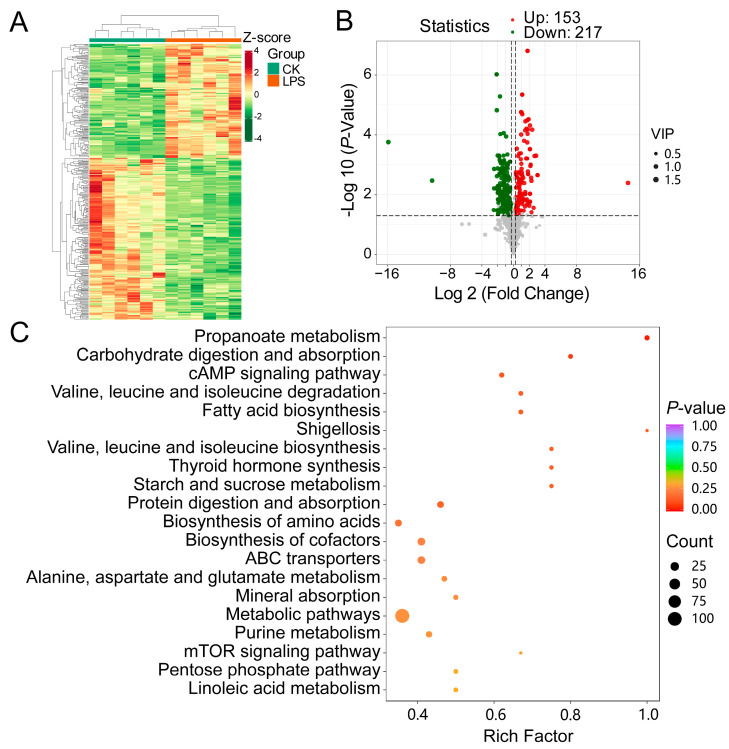
Effects of PASs on metabolic profile of rumen. (**A**) Heat map for differential metabolites from CK group and LPS group. (**B**) Volcano plots of differential metabolites. (**C**) Bubble plot of KEGG enrichment analysis of differential metabolites.

Subsequently, we analyzed the rumen metabolic profiles of the CK and LPS groups using widely targeted metabolomics based on the UPLC-MS/MS detection platform. Here, 943 metabolites were identified and categorized into 17 classes (Figure S6A). Notably, there was a clear separation between the two groups based on the PCA results (Figure S6B). Partial least squares discriminant analysis showed that the metabolic pattern in the sheep rumen changed after PAS addition to the diet (Figure S6C). For model validation, the results showed that it had good stability and reliability (Figure S6D). In addition, the clustering heat map results indicated that metabolites between the two groups exhibited obvious differences (Figure 6A). In this study, 370 DMs were identified, with 153 increased and 217 increased in the LPS group compared with the levels in the CON group (Figure 6B). Detailed information on the identified metabolites is presented in Table S8. An analysis of these DMs showed that the top five classes of compounds were 140 amino acids and their metabolites, 62 organic acids and their derivatives, 33 nucleotides and their metabolites, 28 benzenes and substituted derivatives, and 21 FAs. Subsequently, s KEGG enrichment analysis of the DMs was performed. In total, 122 DMs involved in 119 metabolic pathways were detected in the LPS and CK groups. Propanoate metabolism; carbohydrate digestion and absorption; the cAMP signaling pathway; FA biosynthesis; and valine, leucine, and isoleucine degradation were the top five most significant pathways in the LPS vs. CK group comparison (Figure 6C). Among the metabolites enriched in the aforementioned pathways, the top five metabolites with the largest multiplicative differences were β-alanine, L-lactic acid, succinic acid, serotonin, and 2-hydroxybutanoic acid.

### 3.6. Combined Metagenome and Metabolome Analysis

**Figure 7:**
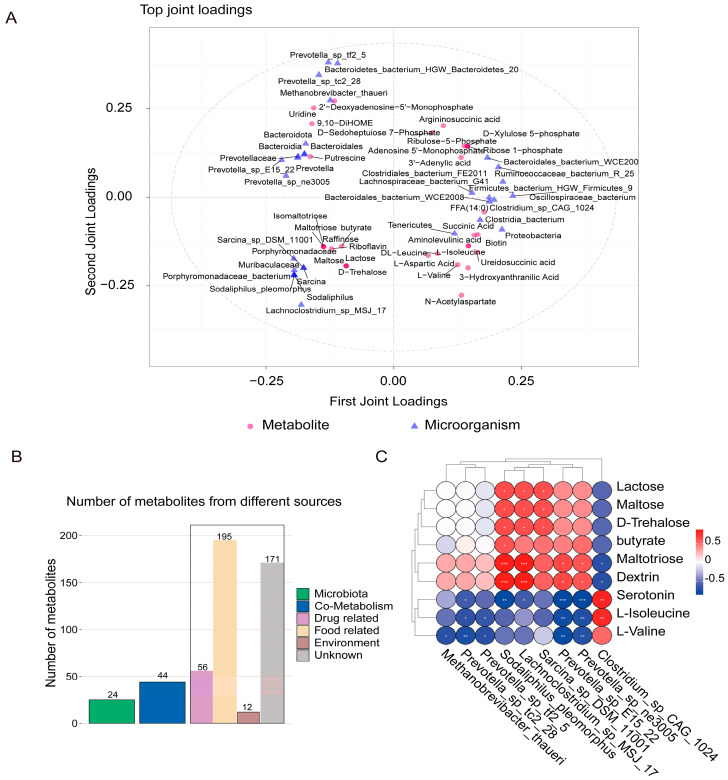
Functional profiles of rumen microbiome and metabolome. (**A**) Metagenome and metabolome O2PLS analysis. (**B**) Bar plot of number of metabolites in different categories. (**C**) Pearson correlation analysis between metabolites and microorganisms. The correlations are indicated by * (0.01 < *p* < 0.05), ** (0.001 < *p* < 0.01), *** (*p* ≤ 0.001), where the red squares indicate positive correlations and blue squares indicate negative correlations.

Subsequently, we performed correlation analyses based on microbial metagenome and metabolomic data, and O2PLS analyses revealed the top 30 most highly associated microbial genera and metabolites (Figure 7A). Also, we excluded host metabolites using the visually interactive online software MetOrigin (version 2.0) (Figure 7B). Subsequently, the top 20 pathways enriched in DMs and the important pathways enriched by microorganisms were compared with propanoate metabolism; FA biosynthesis; starch and sucrose metabolism; and valine, leucine, and isoleucine biosynthesis, pathways shared by metabolites and microorganisms. After analyzing the DMs enriched in the two-omics analysis based on shared pathways, nine DMs were identified, with six metabolites with decreased abundance (lactose, maltose, maltotriose, butyrate, D-trehalose, and dextrin) and three with increased abundance (serotonin, L-isoleucine, and L-valine) used for further analysis. Simultaneously, the common differential microbial genera in the LefSe and Metastats analyses were screened, including eight that were decreased in abundance (*Methanobrevibacter_thaueri*, *Sodaliphilus_pleomorphus*, *Prevotella_sp_E15_22*, *Prevotella_sp_ne3005*, *Prevotella_sp_tc2_28*, *Prevotella_sp_tf2_5*, *Sarcina_sp_DSM_11001*, and *Lachnoclostridium_sp_MSJ_17*) and one microorganism that was increased in abundance (*Clostridium_sp_CAG_1024*). To further explore the relationship between these metabolites and microorganisms, a Pearson correlation analysis was performed. Serotonin, L-isoleucine, and L-valine were positively correlated with *Clostridium_sp_CAG_1024* and negatively correlated with other bacteria. Maltotriose and dextrin were negatively correlated with *Clostridium_sp_CAG_1024* but positively correlated with other genera (*p* < 0.05) (Figure 7C).

### 3.7. Correlation Analysis of Phenotypes with Metabolites and Microorganisms

**Figure 8:**
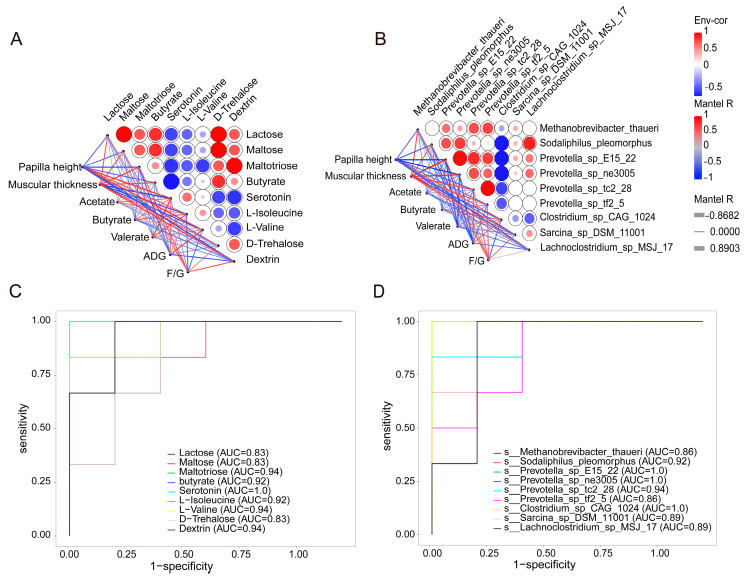
Correlation analysis and prediction analyses based on the random forest model. (**A**) Correlation analysis between metabolites and production performance. (**B**) Correlation analysis between microorganisms and production performance. (**C**) Random forest model used differential metabolites. (**D**) Random forest model used differential species.

To investigate the effects of these microorganisms and metabolites that were screened on rumen epithelial development and growth performance, Pearson’s correlation analysis was performed between them and the phenotypic data. Papillary height was positively correlated with serotonin and L-valine levels (r > 0.60, *p* < 0.05) and negatively correlated with maltotriose and dextrin levels (r < −0.60, *p* < 0.05). Moreover, muscular thickness was negatively correlated with serotonin, L-isoleucine, and L-valine (r < −0.60, *p* < 0.05) and positively correlated with maltotriose, dextrin, and butyrate (r > 0.60, *p* < 0.05). ADG was positively correlated with L-valine (r > 0.60, *p* < 0.05) and negatively correlated with maltotriose and dextrin (r < −0.60, *p* < 0.05) (Figure 8A). In addition, muscular thickness was positively correlated with *Sodaliphilus pleomorphus*, four *Prevotella* strains, *Sarcina_sp_DSM_11001*, and *Methanobrevibacter_thaueri* (r > 0.60, *p* < 0.05) and negatively correlated with *Clostridium_sp_CAG_1024* (r < −0.60, *p* < 0.05). Further, ADG was negatively correlated with several *Prevotella* strains (r < −0.50, *p* < 0.05). *Clostridium_sp_CAG_1024* was positively correlated with papillary height and butyrate levels (r > 0.50, *p* < 0.05) (Figure 8B). Further, we used a random forest model to explore whether phenotypically related DMs and microorganisms could be used to predict effects on rumen epithelial development after PAS treatment. Serotonin, L-valine, and maltotriose were the top three features, with an area under the curve (AUC) > 0.90, for rumen metabolome profiles (Figure 8C). The receiver operating characteristic curve showed that the AUC for *Prevotella*, *S. pleomorphus*, and *Clostridium_sp_CAG_1024* was between 0.86 and 1, indicating that these microorganisms could distinguish between the lambs of the CK and LPS groups (Figure 8D).

## 4. Discussion

In this study, we combined metagenomics and metabolomics to reveal the effects of the dietary supplementation of PASs rich in flavonoids and phenols on the rumen epithelial development and growth performance of lambs. In addition, the effects of rumen microbial composition, function, and metabolites on the rumen fermentation parameters and growth performance of lambs were also studied. At present, studies have shown that adding *Allium mongolicum* Regel and its extracts rich in flavonoids and phenolic compounds to feed can promote the growth performance of sheep and Angus cattle [32,33]. In their study, the authors pointed out that this effect can be attributed to the regulation of phenolic substances on rumen microorganisms, which in turn increases the ADG of animals. In our study, the results were similar to previous reports; that is, the addition of PASs rich in flavonoids and phenols to the diet increased the ADG and decreased the F/G ratio of sheep. The reason for the above results may be related to the flavonoids in PASs, which have anti-inflammatory and antioxidant properties. They can improve the digestion ability of ruminants by maintaining the integrity of the intestinal barrier to improve feed conversion efficiency [34]. This study confirmed that the rumen contents of acetic acid, propionic acid, and butyric acid increased after the addition of PASs rich in flavonoids, consistent with the results of Balcells et al. They pointed out that supplementation with flavonoid extracts could improve rumen fermentation parameters and relieve the signs of rumen acidosis [35]. As a large fermenter, the rumen produces high concentrations of FAs, which can stimulate the proliferation and development of the rumen epithelium [36,37]. Of note, in the present study, PAS addition to the diet increased the height and width of the rumen papillae, which could be closely related to the increased concentration of FAs in the rumen. These results indicate that flavonoid-rich PASs might promote the development of the rumen epithelium by improving rumen fermentation parameters.

The rumen microbial community is composed of complex and diverse microorganisms, including bacteria, protozoa, archaea, fungi, and viruses. The results of this study showed that the microbial composition of bacteria, archaea, and viruses changed significantly in the rumens of both groups after PAS addition to the diet. In terms of quantity, bacteria are the dominant species in the rumen and play an essential role in digestion, fermentation, and degradation, suggesting that they have a more important role in the growth and nutrient absorption of the host than other microorganisms [38]. Notably, those of *Prevotella* were the predominant less abundant species in the LPS group. *Prevotella* decomposes complex carbohydrates and promotes the production of acetic acid, lactate, and succinic acid during metabolic processes [39]. Previous studies have shown that *Prevotella* and *Muribaculaceae* accumulation in the intestine aggravates intestinal inflammation [39]. Moreover, young apple polyphenols can inhibit the activity of *Prevotella* by altering its morphological changes [40]. This reduced activity could be due to polyphenols altering the hydrophobicity of the cell membrane, leading to the separation and eventual rupture of the cell wall and cell membrane, which in turn produces irreversible changes in the intracellular composition [41]. Our results suggest that the decrease in the relative abundance of *Prevotella* in the rumen after PAS addition to the diet might have a strong relationship with the phenolic compounds rich in PASs. Archaea are the main methane-producing bacteria in the rumen ecosystem and can use the hydrogen produced in metabolic processes to maintain the internal environmental homeostasis of microbial fermentation [42]. A decrease in the abundance of *Methanococcus* positively affects the rumen microbial community by directly reducing methane production, changing rumen fermentation patterns, increasing propionic acid production, and promoting the growth of beneficial bacteria [43]. As a functional substance, flavonoids can inhibit the growth of harmful bacteria in the gastrointestinal tract by destroying the lipid bilayer of harmful bacteria or affecting the permeability of their cell membranes and providing metabolic substrates for intestinal flora to promote the growth of beneficial bacteria, thereby resulting in the optimization of the intestinal flora structure. In this study, the abundance of *Methanococcus vannielii* was significantly reduced in the LPS group, which could be because the flavonoids in PASs disrupted the permeability of the cell membrane, thereby inhibiting bacterial activity and resulting in changes in the abundance of flora. Rumen viruses are highly diverse and can infect different rumen microorganisms, including methanogens. They also regulate the composition of their microbial hosts by lysing ruminal microorganisms at different trophic levels, thereby improving the circulation of nutrients and microbial proteins [44]. Our results showed that after PAS addition to the diet, the abundance of most viruses among the three taxonomic classes, including phyla and species, was lower than that in the CK group. We speculated that some active components of PASs might have bacteriostatic and detoxification effects; however, the specific mechanisms require further study.

The function of the rumen microbiome is more conserved compared to the taxonomic differences between two groups of animals [45,46]. Carbohydrate metabolism plays a crucial role in rumen microbial communities; it is involved in energy acquisition and partitioning via a variety of metabolic pathways and affects host physiology [47]. Notably, KEGG functional enrichment analysis revealed that carbohydrate degradation functions, including starch and sucrose metabolism, propanoate metabolism, and downstream pathways used to convert glucose to pyruvate, were significantly enriched in the rumens of sheep of the CK group, suggesting that the microorganisms in the CK group have a higher catabolic capacity for carbohydrates and might produce more pyruvate and other hydrolysates. An analysis of genes encoding enzymes related to starch and sucrose metabolism and downstream pyruvate metabolism pathways revealed that those involved in methanogenesis were more abundant in the rumen microbes of the CK group. For example, EC 2.8.4.1, also known as methyl coenzyme M reductase, catalyzes methane production in the final step of the methane metabolism process [48]. Our results suggest that the main energy, in the form of VFA sources during the fermentation of rumen microorganisms, could be reduced in the CK group because the reactions of a series of methanogenic bacteria, catalyzed by proteins involved in methanogenesis, utilize the hydrogen and carbon dioxide produced during the fermentation of VFAs for methane production. From these results, we can conclude that the microorganisms in the CK group might have provided more pyruvate, owing to a higher level of substrate degradation, but VFA production via pyruvate might not have played a corresponding functional role, compared to that of the rumen microorganisms in the LPS group, because the hydrolysis products were ultimately presented in the form of methane. Compared to those in the CK group, the VFA concentrations were higher in the rumen of sheep in the LPS group, indicating that LPS group rumen microorganisms can use the hydrolysate more efficiently to produce VFAs, in turn providing more energy and nutrients for epithelial development and growth performance. In future studies, it will be necessary to further identify rumen development indicators and assess methane emissions to verify our hypothesis, in addition to determining the functions and groups of active microorganisms, to determine their roles in related metabolic pathways.

In addition to the important role of carbohydrate anabolism in the rumen flora, nitrogen metabolism also plays an active role in rumen microorganisms, and this can regulate the feed utilization efficiency of ruminants [45]. Shabat et al. reported that pathways related to nitrogen metabolism are closely related to feed efficiency [49]. In particular, valine, leucine, and isoleucine degradation; lysine degradation; and histidine metabolism are significantly enriched in microorganisms of dairy cows with low feed efficiency. In addition, branched-chain amino acids (BCAAs) play an important role in microbial protein synthesis, including L-valine, L-leucine and L-isoleucine, which can improve production efficiency through metabolic pathways [50]. Our results showed that the BCAA biodegradation function in the CK group indicated that less microbial protein may be produced in the CK group, resulting in more inefficient feed conversion efficiency. In contrast, LPS may have a higher feed conversion efficiency. The above results showed that the addition of PASs might improve feed efficiency in sheep.

In this study, the metagenomic results revealed differences in the compositions and functions of the two groups of microorganisms. Subsequently, we analyzed the rumen metabolome and found that metabolic patterns differed between the two groups. Of note, the rumen metabolome and metagenomic data were in good agreement; that is, the common differential pathways were mainly involved in carbohydrate anabolism and amino acid and FA metabolism. Among them, phosphate and phosphite metabolism and purine metabolism pathways were upregulated, whereas starch and sucrose metabolism; propanoate metabolism; FA biosynthesis; and valine, leucine, and isoleucine biosynthesis pathways were downregulated. Subsequently, the differential metabolites enriched in the aforementioned pathways were screened. Among them, the levels of three metabolites, namely serotonin, L-isoleucine, and L-valine, were increased in the LPS group. Serotonin is a metabolite produced by intestinal microorganisms (such as *Clostridium sporogenes* and *Ruminococcus gnavus*) to break down tryptophan. It can accelerate the transport of nutrients in the gastrointestinal tract by activating 5-hydroxytryptamine receptor 4 and increasing anion-dependent fluid secretion in the proximal colon of mice [51]. In addition, the gut microbiota can regulate the function of the immune system by modulating the production of serotonin in the gut and interacting with intestinal immune cells [52]. The metabolism of L-isoleucine and L-valine could help maintain the stability and health of the rumen microbial community by promoting protein synthesis and energy production [53]. In addition, they can serve as growth factors for rumen cellulose-decomposing bacteria, which generally have a positive effect on rumen microbial fermentation and improved feed efficiency [54]. Pearson’s correlation analysis showed that these three metabolites were positively correlated with *Clostridium* and negatively correlated with *Prevotella* and *Methanobrevibacter*. These results indicate that dietary PAS supplementation could have a positive effect on rumen development and health by changing the composition of beneficial microorganisms and their metabolic processes.

Finally, we analyzed the relationship between rumen microbial genera or metabolites and the host phenotype. Here, papilla height was positively correlated with metabolites such as serotonin, L-isoleucine, and L-valine. Rumen microbial metabolites, such as indole-3-carboxaldehyde and prostaglandin D2, promote the development of the rumen epithelium. Moreover, these metabolites promote rumen epithelial and muscular layer development by activating specific signaling pathways, such as the Wnt/β-catenin and Ca^2+^ signaling pathways [50]. VFAs are chemical stimulants involved in morphological development, and they also increase the absorption capacity of rumen tissues, with butyric acid having the most significant stimulatory effect on the rumen epithelium [55]. In this study, PAS addition to the diet promoted the development of rumen papillae, which could be closely related to an increase in the butyric acid content in the rumen. However, no direct evidence indicating that isoleucine and valine promote the proliferation and differentiation of ruminal epithelial cells exists. We speculate that they can indirectly affect rumen development by modulating rumen epithelial cell absorption and metabolism. In addition, several *Prevotella* species are positively correlated with rumen muscle thickness. Further, it plays an important role in nutrient metabolism, such as the degradation of cellulose and hemicellulose, the synthesis of proteins, and the metabolism of carbohydrates [56,57,58]. We speculate that this might promote the healthy development of the rumen muscle layer by improving the efficiency of rumen digestion and the utilization of nutrients. Further, *Clostridium_sp_CAG_1024* was positively correlated with the concentration of butyric acid. *Clostridium* is a key bacterium involved in butyric acid metabolism. It also consists of the dominant microbial species involved in the microbial fermentation process, and it promotes butyric acid [59]. *Clostridium_sp_CAG_1024*, as a member of the *Clostridium* genus, might also have a positive effect on the production of butyric acid.

High-throughput sequencing technology and machine learning have been developed and used to better understand the role of the microbiota and microbial metabolites in host phenotypes, health, and disease [60,61]. In this study, we found that rumen microorganisms and metabolites could be used to predict rumen epithelium development. Of note, the random forest model showed that serotonin, L-valine, and maltotriose could be used as key metabolic markers to distinguish the rumen of sheep treated with PASs, with an accuracy of more than 92%. At the same time, *Prevotella* (*Prevotella_sp_E15_22* and *Prevotella_sp_ne3005*) and *Clostridium* (*Clostridium_sp_CAG_1024*) could be used as key microbial markers to distinguish sheep rumens after the administration of a PAS diet, with an accuracy of 100%. These data only represent models for machine learning prediction; the use of early microbial markers to predict sheep production performance in actual production practice is still undefined and challenging, and more production data are needed to integrate and analyze the best biomarkers and use them in future production practices.

## 5. Conclusions

Dietary PAS supplementation could increase the height and width of the rumen papilla; increase acetic, butyric, and propionic acid concentrations; improve the average daily gain; and reduce the F/G ratio in sheep. Compared with that in the CK group, PAS dietary supplementation improved the abundance of specific flora; specifically, it increased the abundance of *Clostridiales* and *Bacteroidales* and reduced the abundance of *Prevotella*, *Butyrivibrio*, and *Methanococcus*. In addition, energy utilization-related pathways such as carbohydrate synthesis and nitrogen metabolism were upregulated, and the concentrations of metabolites such as L-isoleucine, serotonin, and L-valine were increased. In summary, the results of this study are consistent with our hypothesis that the addition of PASs rich in flavonoids and polyphenols to feed can improve rumen epithelial development and growth performance through the combined action of rumen microorganisms and metabolites. Therefore, PASs could be developed as an unconventional feed resource and applied to sheep production practices to promote rumen epithelial development and growth performance.

## Figures and Tables

**Table 1:** Effects of dietary PAS supplementation on growth performance of Hu sheep.

Item	Group ^1^			*p* Value
	CK	LPS	HPS	
Initial body weight (kg)	26.31 ± 0.94	24.40 ± 1.25	25.15 ± 1.33	0.532
Final body weight (kg)	37.25 ± 1.42	37.88 ± 1.51	38.07 ± 1.27	0.912
Average daily gain (g/d)	121.48 ± 7.67 ^b^	149.82 ± 6.29 ^a^	143.52 ± 5.40 ^a^	0.019
Average daily feed intake (g/d)	1180.03 ± 86.18	1151.35 ± 91.96	1139.57 ± 85.58	0.946
Carcass weight (kg)	18.80 ± 0.75	18.51 ± 0.54	19.80 ± 0.72	0.395
F/G ^2^	9.71 ± 0.71 ^a^	7.68 ± 0.61 ^b^	7.94 ± 0.60 ^ab^	0.047

^1^ CK, basal diet; LPS, 3% prickly ash seeds were used to replace the same amount of roughage; HPS, 6% prickly ash seeds were used to replace the same amount of roughage. ^2^ F/G, average daily feed intake/average daily gain ratio. ^a,b^ means within a row with different superscript letters are significantly different (*p* < 0.05).

**Table 2:** Effects of PASs on rumen fermentation parameters in lambs.

Parameters	Group ^1^			*p* Value
	CK	LPS	HPS	
pH	6.26 ± 0.06	6.41 ± 0.03	6.37 ± 0.03	0.052
NH_3_-N (mg/dL)	11.85 ± 0.13	12.02 ± 0.18	11.83 ± 0.13	0.639
Acetate (mmol/L)	53.33 ± 0.76 ^b^	56.54 ± 0.98 ^a^	50.85 ± 1.05 ^b^	<0.01
Propionate (mmol/L)	14.14 ± 0.47 ^b^	13.66 ± 0.56 ^b^	17.22 ± 0.47 ^a^	<0.01
Iso-butyrate (mmol/L)	0.72 ± 0.02	0.68 ± 0.01	0.72 ± 0.04	0.474
Butyrate (mmol/L)	10.44 ± 0.33 ^b^	11.37 ± 0.33 ^a^	10.16 ± 0.21 ^b^	0.027
Iso-valerate (mmol/L)	0.75 ± 0.04	0.79 ± 0.02	0.72 ± 0.03	0.191
Valerate (mmol/L)	0.63 ± 0.02 ^b^	0.76 ± 0.03 ^a^	0.64 ± 0.03 ^b^	0.008
Acetate/propionate	3.79 ± 0.14 ^a^	4.18 ± 0.25 ^a^	2.96 ± 0.09 ^b^	<0.01
Total VFAs ^2^ (mmol/L)	80.01 ± 1.19	83.81 ± 0.58	80.31 ± 1.40	0.177

^1^ CK, basal diet; LPS, 3% prickly ash seeds were used to replace the same amount of roughage; HPS, 6% prickly ash seeds were used to replace the same amount of roughage. ^2^ Total VFAs, total volatile fatty acids. ^a,b^ means within a row with different superscript letters are significantly different (*p* < 0.05).

## Data Availability

The metagenomic data were deposited in the NCBI SRA Database (PRJNA1142090). Other data are contained within this article and Supplementary Materials.

## Supplementary Materials

The following supporting information can be downloaded at https://www.mdpi.com/article/10.3390/microorganisms12112242/s1, Figure S1: Classification of bioactive substances in prickly ash seeds; Figure S2: PcoA; Figure S3: Microbial compositional profiles of CK and LPS sheep; Figure S4: Comparison of viruses’ phylum, genus, and species; Figure S5: Functional analysis of rumen flora. (A) PCoA diagram of KEGG functional units. (B) Abundance of KEGG function in CK and LPS groups; Figure S6: Multivariate analyses of rumen metabolites. (A) Metabolite classification. (B) PCA score plot. (C) OPLS-DA analysis of metabolites in rumen. (D) Permutation testing of OPLS-DA in CK vs. LPS groups; Table S1: Content of conventional nutrients in prickly ash seeds; Table S2: Composition and nutrient levels of basal diets (DM basis); Table S3: Summary of sequence data generated from rumen samples of sheep; Table S4: Comparison of bacterial phylum, genus, and species; Table S5: Comparison of archaea’s phylum, genus, and species; Table S6: Comparison of viruses’ phylum, genus, and species; Table S7: The KEGG pathways enrichment analyses of rumen flora in the CK and LPS groups; Table S8: Details for all differential metabolites in each group.
