# Supplementary material for: Prickly Ash Seeds Improve the Ruminal Epithelial Development and Growth Performance of Hu Sheep by Modulating the Rumen Microbiota and Metabolome
Source: Microorganisms. 2024 Nov 6;12(11):2242. doi: 10.3390/microorganisms12112242 (PMC11596069; doi:10.3390/microorganisms12112242)
Supplement: Supplementary file 1 [file microorganisms-12-02242-s001.zip › Supplementary figure.pdf]

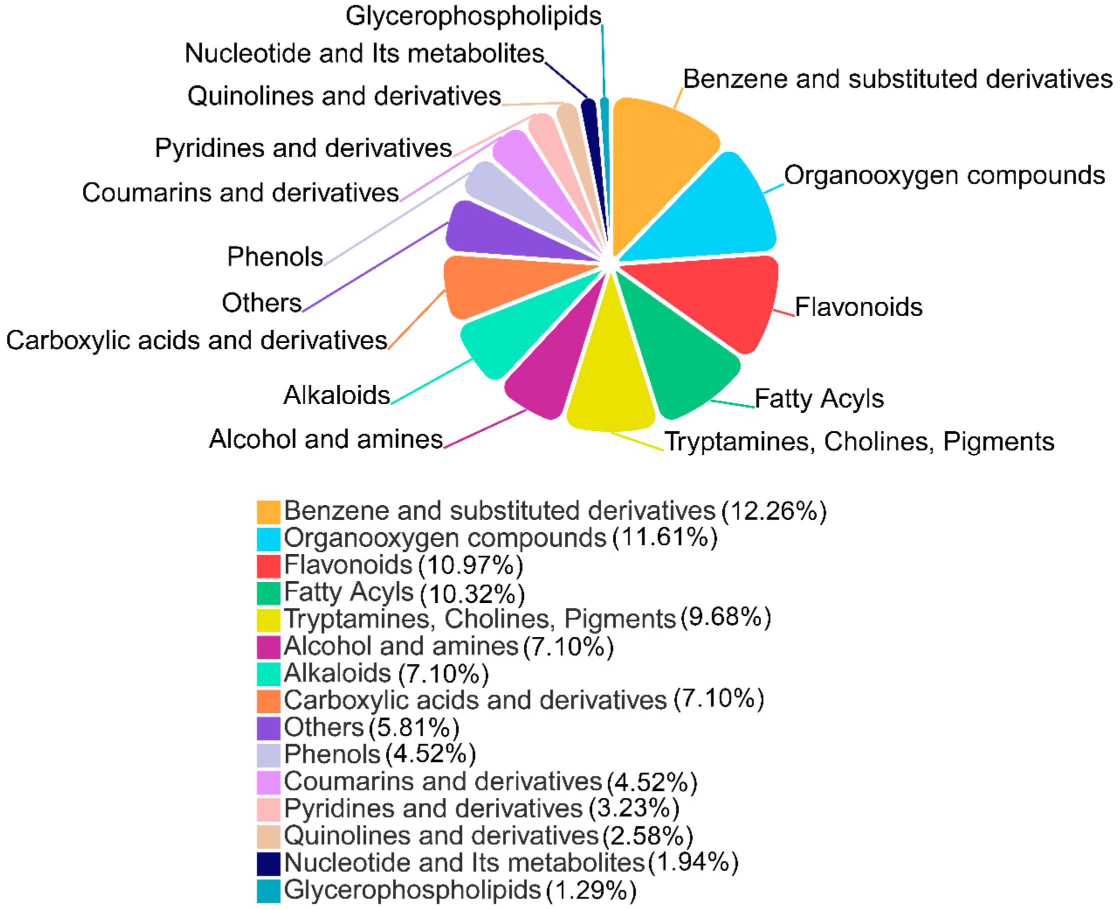

Figure S1. Classification of bioactive substances in Prickly ash seed

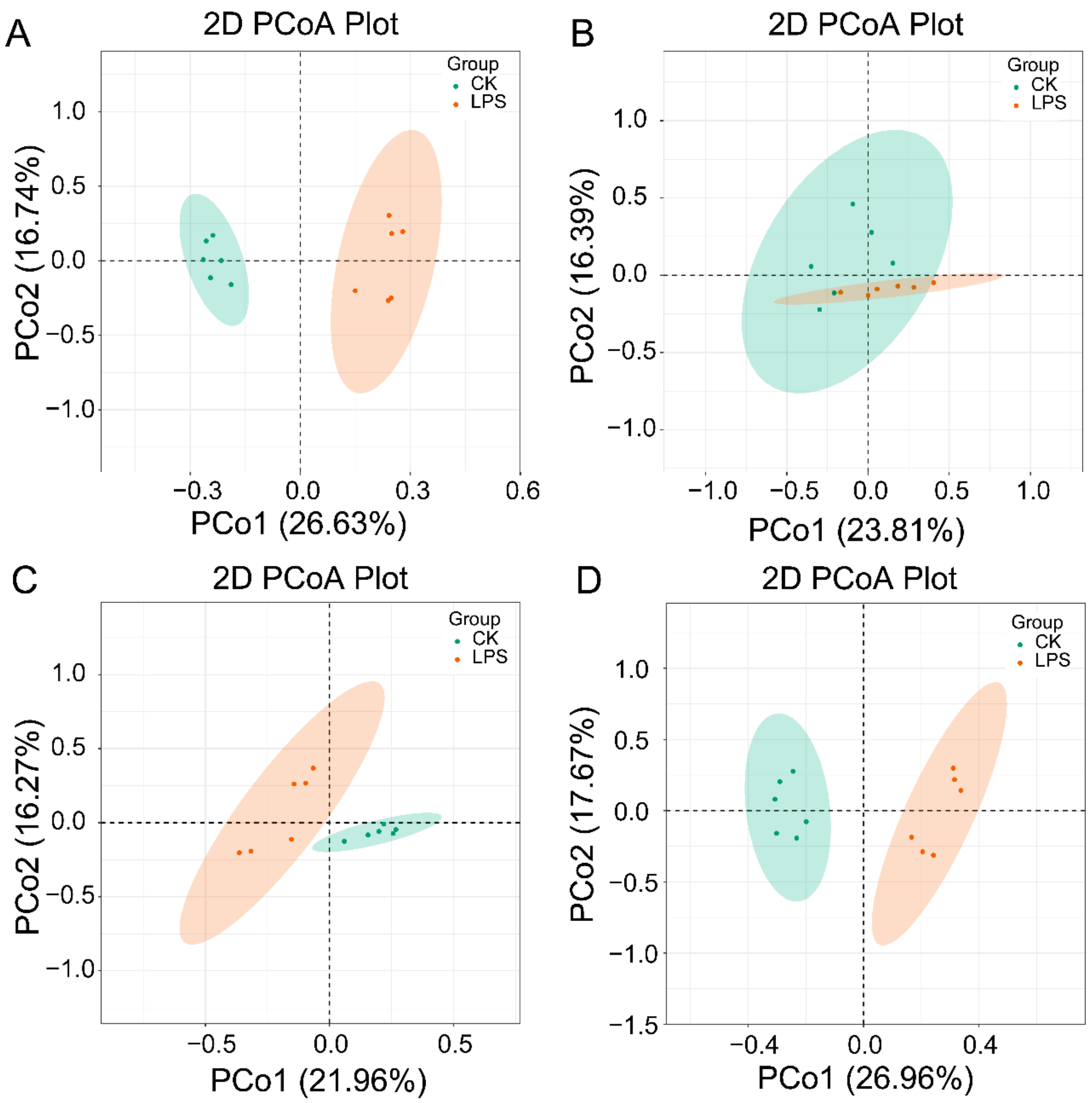

Figure S2. PcoA analysis. (A) 2D PcoA plot of bacteria. (B) 2D PcoA plot of archaea. (C) 2D PcoA plot of eukaryota. (D) 2D PcoA plot of viruses.

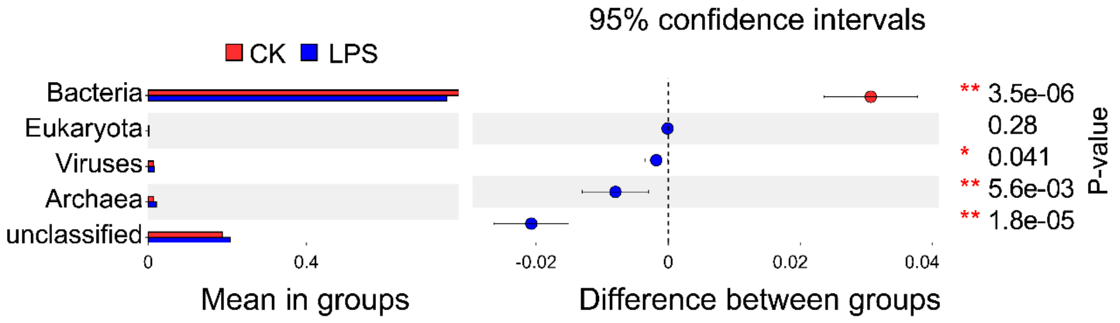

Figure S3. Microbial compositional profiles of CK and LPS sheep.

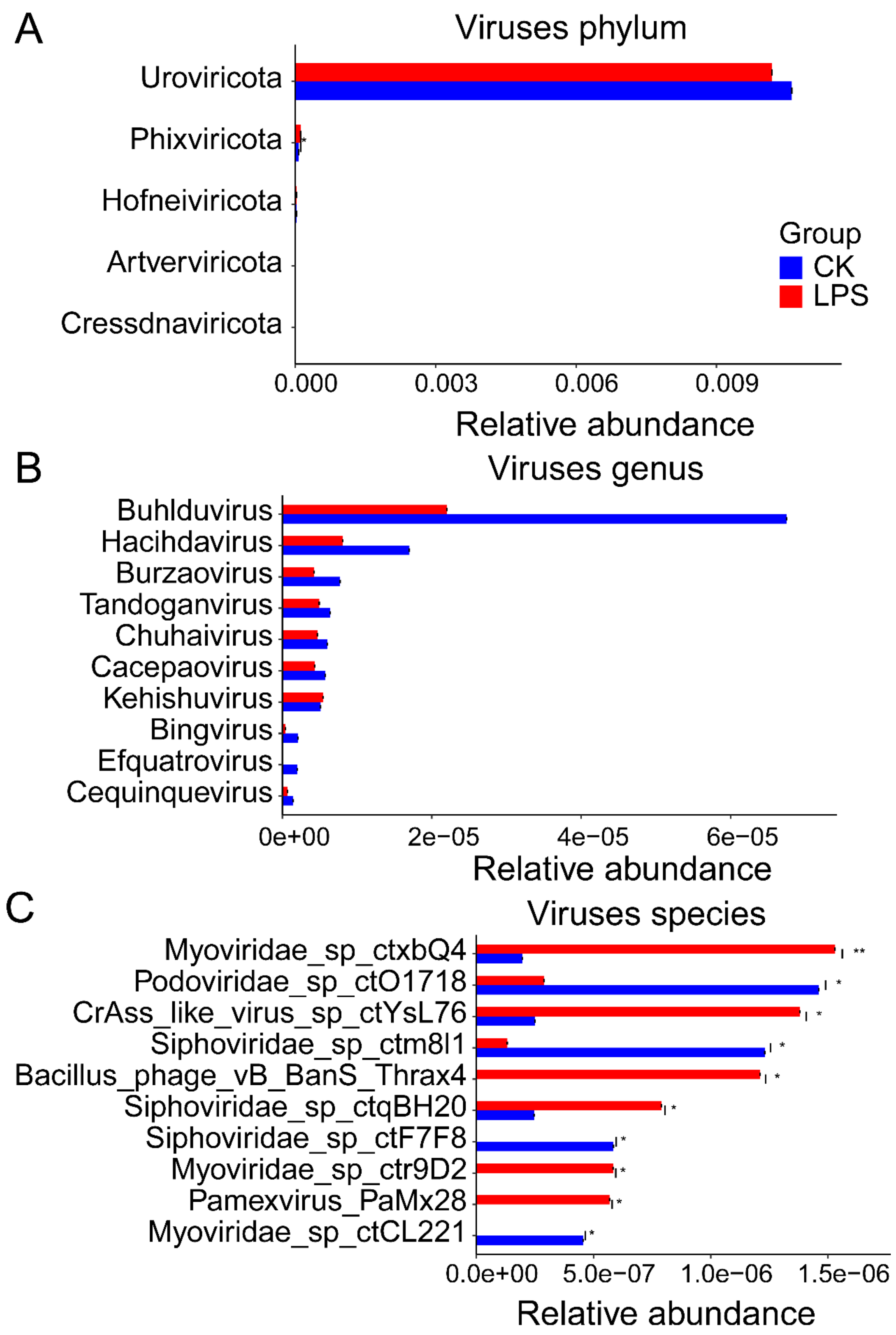

Figure S4. Comparison of viruses phylum, genus, and species.

(A) Viruses phylum were tested by Metastats analysis. (B) Viruses genus were tested by Metastats analysis. (C) Viruses species were tested by Metastats analysis.

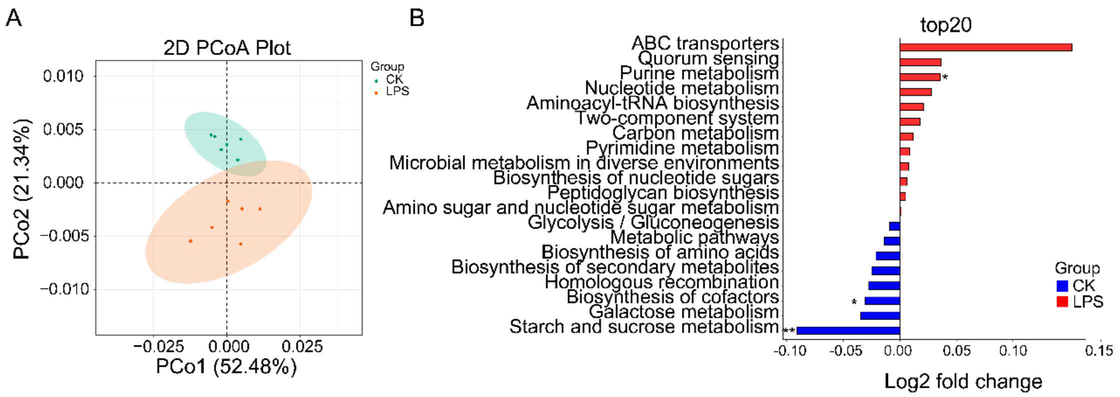

Figure S5. Functional analysis of rumen flora.

(A) PCoA diagram of KEGG functional units. (B) The abundance of KEGG function in the CK and LPS.

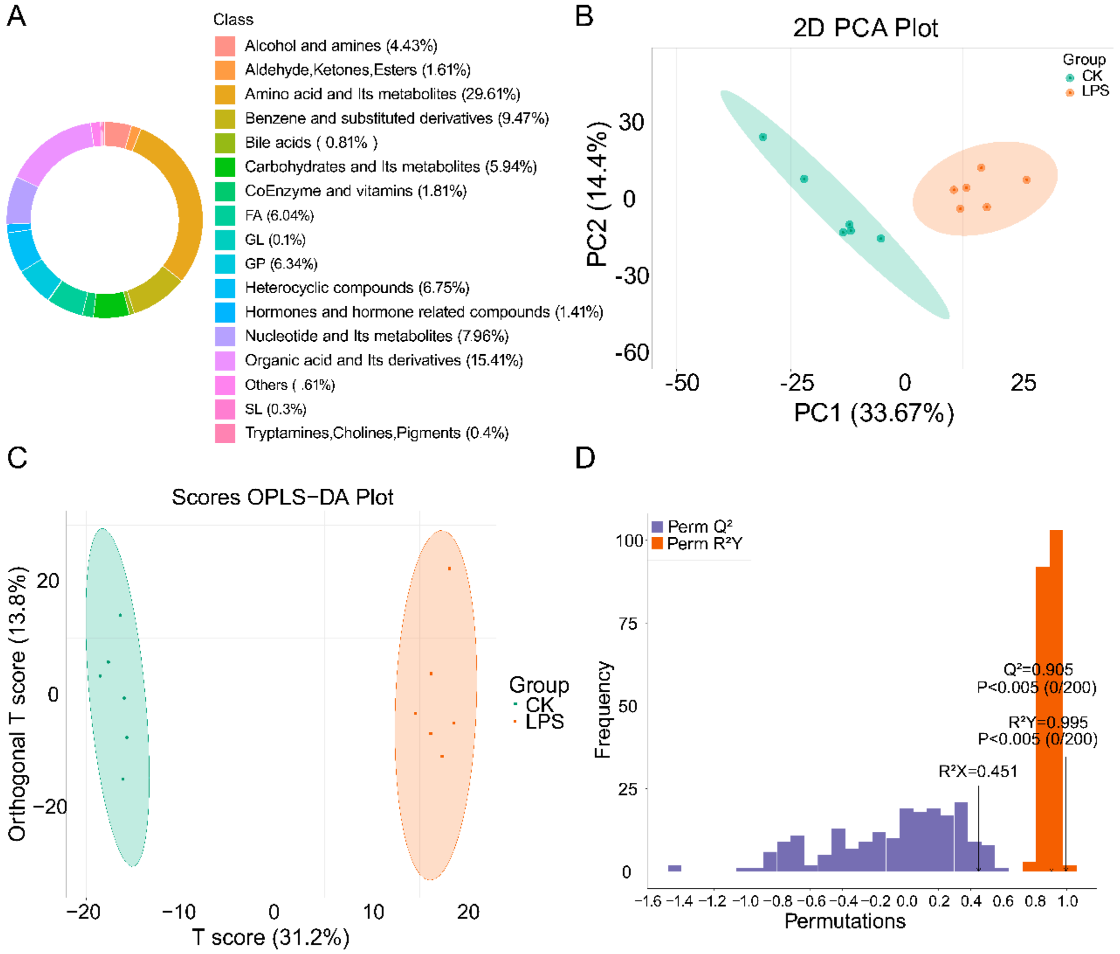

Figure S6. Multivariate analyses of rumen metabolites.

(A) Metabolite classification. (B) PCA score plot. (C) OPLS-DA analysis of metabolites in rumen. (D) Permutation testing of OPLS-DA in CK vs. LPS group.
